# Evaluation of Laser-Assisted Trans-Nail Drug Delivery with Optical Coherence Tomography

**DOI:** 10.3390/s16122111

**Published:** 2016-12-12

**Authors:** Meng-Tsan Tsai, Ting-Yen Tsai, Su-Chin Shen, Chau Yee Ng, Ya-Ju Lee, Jiann-Der Lee, Chih-Hsun Yang

**Affiliations:** 1 Department of Electrical Engineering, Chang Gung University, Taoyuan 33302, Taiwan; mengtsan@gmail.com (M.-T.T.); trendy1991818@gmail.com (T.-Y.T.); jdlee@mail.cgu.edu.tw (J.-D.L.); 2 Medical Imaging Research Center, Institute for Radiological Research, Chang Gung University and Chang Gung Memorial Hospital at Linkou, Taoyuan 33302, Taiwan; 3 Department of Dermatology, Chang Gung Memorial Hospital, Linkou 33305, Taiwan; charlene870811@gmail.com; 4 Department of Ophthalmology, Chang Gung Memorial Hospital, Linkou 33305, Taiwan; suchin@adm.cgmh.org.tw; 5 College of Medicine, Chang Gung University, Taoyuan 33302, Taiwan; 6 Institute of Electro-Optical Science and Technology, National Taiwan Normal University, Taipei 11677, Taiwan; yajulee@ntnu.edu.tw; 7 Department of Neurosurgery, Chang Gung Memorial Hospital, LinKou 33305, Taiwan

**Keywords:** drug delivery, nail, optical coherence tomography, fractional laser, laser ablation

## Abstract

The nail provides a functional protection to the fingertips and surrounding tissue from external injuries. The nail plate consists of three layers including dorsal, intermediate, and ventral layers. The dorsal layer consists of compact, hard keratins, limiting topical drug delivery through the nail. In this study, we investigate the application of fractional CO_2_ laser that produces arrays of microthermal ablation zones (MAZs) to facilitate drug delivery in the nails. We utilized optical coherence tomography (OCT) for real-time monitoring of the laser–skin tissue interaction, sparing the patient from an invasive surgical sampling procedure. The time-dependent OCT intensity variance was used to observe drug diffusion through an induced MAZ array. Subsequently, nails were treated with cream and liquid topical drugs to investigate the feasibility and diffusion efficacy of laser-assisted drug delivery. Our results show that fractional CO_2_ laser improves the effectiveness of topical drug delivery in the nail plate and that OCT could potentially be used for in vivo monitoring of the depth of laser penetration as well as real-time observations of drug delivery.

## 1. Introduction

The nail is a modified form of stratum corneum, with a thick laminated keratinized structure overlying the nail bed and matrix. However, the thick structure limits drug delivery to the nail bed, which is problematic when it comes to treating nail diseases such as onychomycosis. The nail plate is composed of 25 sheets of keratinized cells that can be divided into dorsal, intermediate, and ventral layers. Compared with the intermediate layer, the dorsal and ventral layers are thinner. The dorsal and ventral layers consist of harder skin-type keratin with lipids. In contrast, the intermediate layer is composed of hair-type keratin with few lipids, making the intermediate layer more flexible. Therefore, the dorsal layer forms a barrier for drug delivery [1,2,3,4]. To improve the efficiency of drug delivery through the nail, a new strategy is to produce micropores on the nail to remove the dorsal layer [5,6]. Therefore, the development of permeation-enhanced techniques for skin has become an important area of study to improve drug delivery. Recently, transdermal drug delivery became a new route of drug and vaccine administration, providing the advantages of avoiding the first-pass metabolism, sustained therapeutic action, and better patient compliance [7,8]. Strategies to bypass the tightly packed stratum corneum, the rate-limiting step in transdermal drug penetration, will facilitate topical medication delivery deep into the skin. Several methods have been developed to improve transdermal drug delivery, including chemical enhancers [9,10], nanocarriers [11,12], microneedles [13,14], sonophoresis [15,16], and iontophoresis [17,18]. The biocompatibility and biotoxicity of chemical enhancers and nanocarriers are important issues. For microneedles, although new biodegradable polymers reduce the risk of microneedles retained in skin tissue, these materials are not able to produce sufficient mechanical strength to penetrate the skin barrier. On the contrary, metallic microneedles can easily penetrate the skin barrier but may cause allergic reactions. Both ultrasound and iontophoresis to facilitate drug delivery have been proposed in previous studies, but accurate control of the treatment depth remains a challenging issue.

The development of laser techniques has promoted various applications, in particular for therapies and biomedical imaging. In therapeutic applications, lasers offer an excellent solution in clinical medicine because they result in less bleeding, reduced infections, and minimized incision areas [19]. With a pulsed high-energy laser, the biological tissue can be coagulated, and even ablated, which enables skin tightening, hemangioma treatment, and the removal of unwanted hair and blood vessels [20,21,22,23,24]. Ablative fractional lasers are primarily used to treat photodamaged skin, deep rhytides and scarring. Current fractional laser systems for dermatology include carbon dioxide (CO_2_, 10,600 nm) and erbium-doped yttrium aluminum garnet (Er:YAG, 2940 nm) lasers. The fractional CO_2_ laser produces deep vertical holes down to the dermis to assist the delivery of topically applied drugs into the skin. Recently, this approach was used in the treatment of fungal nail diseases [25]. The micro-channel array created by a fractional CO_2_ laser creates tiny pores on the skin surface that enhance the penetration of topically applied drugs. Penetration-enhanced techniques for skin and nails are rapidly developing, enabling significant increases in the efficiency of disease treatment.

Currently, various optical imaging approaches to monitor transdermal drug delivery have been proposed, such as confocal laser scanning microscopy (CLSM) [26], two-photon microscopy (TPM) [27], infrared microscopic imaging (IMI) [28], and Raman microscopy (RM) [28]. Although both CLSM and TPM can provide cellular-level resolution, their imaging depth is limited to hundreds of micrometers, which is not deep enough to observe drug diffusion beneath the skin surface. Moreover, CLSM or TPM need extra fluorescent labeling. Compared to CLSM and TPM, IMI provides a wider imaging field, but skin specimens must be carefully prepared before imaging, and IMI cannot be used for in vivo imaging. The imaging depth of RM is limited when used for studies on drug delivery. The thickness of nails ranges from hundreds of micrometers to several millimeters, making the approaches mentioned earlier unsuitable for investigating drug delivery via nails. Moreover, these methods do not acquire depth and time-resolved information of the dynamics of drug diffusion from the nail surface to the nail bed. Therefore, in this paper, we propose the use of optical coherence tomography (OCT) to investigate the dynamics of transdermal drug delivery.

OCT uses backscattered tissue signals to reconstruct the 2D/3D morphology of biological tissue [29,30,31]. Compared to ultrasound imaging, OCT provides higher resolutions in both the transverse and depth directions (up to 1–10 μm). Moreover, OCT can probe deeper tissue structures than of microscopic techniques such as confocal microscopy, TPM, and harmonic generation microscopy [32,33,34]. Besides this deeper imaging depth, OCT imaging is noninvasive, has a high imaging speed, and can be used for internal hollow organ scanning with concomitant use of an endoscope. Various functional OCT with different purposes have been developed including optical coherence angiography [35,36], polarization-sensitive OCT for the measurement of tissue birefringence [37,38], and optical coherence elastography [39,40]. In previous studies, we have demonstrated that the photothermolysis of human skin induced by an ablative laser can be monitored with OCT [41]. Furthermore, preliminary OCT results have proven the feasibility of laser-assisted therapy [42]. In this study, we investigate the time-dependent variation of OCT intensity during the diffusion process of drug particles. Additionally, we also estimate the time-dependent speckle variance (SV) [43,44,45,46] of OCT intensity, observe in vivo laser-assisted drug delivery, and evaluate the diffusion ability of different drug preparations (liquid and cream drugs) in nails treated with fractional CO_2_ laser. Finally, we evaluate the relative diffusion velocities of cream and liquid drugs in the nail by estimating the center-of-mass locations of time-dependent SV.

## 2. Experiment Method and Setup

The experiments in this study were approved by the Chang Gung Medical Foundation Institutional Review Board (No. 101-2921A3) and were conducted in the outpatient clinic of the Department of Dermatology of Chang Gung Memorial Hospital, Taipei, Taiwan. The volunteers were subjected to irradiance by a fractional CO_2_ laser (UltraPulse Encore Active FXTM; Lumenis, Santa Clara, CA, USA) under various exposure energies of 20, 30, 40, and 50 mJ. The average power and the pulse width of the used CO_2_ laser are 330 W and 0.15 ms, respectively. Single laser pulse induced each MAZ on the nail plate. The maximum output energy was up to 50 mJ. The fingernails of the volunteers were exposed to laser energies of 20, 30, 40, and 50 mJ. Fingernails were scanned by the OCT system after laser exposure to discern induced photothermolysis. Liquid or cream topical drugs (Sulconazole Nitrate) were then applied to the exposed region of the fingernail, and we scanned the nail continuously with OCT. The liquid drug we used was an Exelderm solution consisting of Sulconazole nitrate with a concentration of 1%, and the cream drug we used was Exelderm cream composed of Sulconazole nitrate with a concentration of 1%. The Exelderm solution is a solution of propylene glycol, poloxamer 407, polysorbate 20, butylated hydroxyanisole, and purified water, with sodium hydroxide. Exelderm cream is in an emollient cream base, which consists of propylene glycol, stearyl alcohol, isopropyl myristate, cetyl alcohol, polysorbate 60, sorbitan monostearate, glyceryl stearate and PEG-100 stearate, ascorbyl palmitate, and purified water with sodium hydroxide. Additionally, previous reports have demonstrated that propylene glycol is a drug load enhancer and that sodium hydroxide is an uptake rate enhancer [47]. Before OCT measurement, the finger was immersed into the ultrasonic cleaner to remove the dust in microthermal ablation zones (MAZs) for 5 min and then dried in air for 30 min.

In this study, a swept-source OCT (SS-OCT) system was set up for in vivo fingernail scanning. The setup of the SS-OCT system is similar to that of a previous study [42]. A swept source (HSL-20, Santec Corp., Aichi, Japan) at 1.3 μm was used as the light source of the OCT system with a scanning spectrum of 110 nm. The longitudinal and transverse resolutions are approximately 7 and 5 μm, respectively. The physical scanning range is 3 × 3 × 3 mm^3^. The maximum imaging depth of this OCT system is approximately 3 mm. Because the light source can provide a scan rate of 100 kHz, the corresponding frame rate of the OCT system was set to 100 frames/s. Unconscious motion by the volunteer during the OCT measurement was reduced using a specially designed mount fabricated by a 3D printer to fix the finger stably. Moreover, to investigate the feasibility of laser-assisted drug diffusion, the drug was rubbed on the nails and scanned with OCT. We record sequential 2D OCT images before and after the drug application.

## 3. Results and Discussion

**Figure 1:**
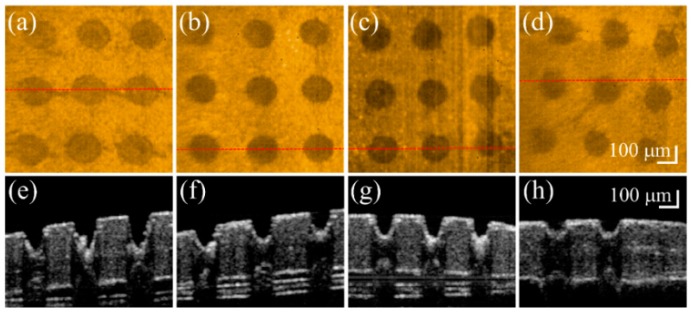
In vivo (**a**–**d**) top-view and (**e**–**h**) representative cross-sectional OCT images of four fingernails after fractional laser exposures to (from left to right) energies of 50, 40, 30, and 20 mJ. The red-dash lines in (**a**–**d**) indicate the corresponding locations of (**e**–**h**).

Fractional laser ablation causes tissue vaporization, producing a microthermal ablation zone (MAZ) array. However, the induced MAZ penetration depth is hard to predict because of differences in the optical properties of biological tissues. To investigate the induced photothermolysis on the nail, four fingernails of a 26-year-old volunteer were sequentially exposed to fractional CO_2_ laser with exposure energies of 50, 40, 30, and 20 mJ. The four treated nails were then scanned in vivo by the OCT system to acquire 3D microstructural images. Figure 1 shows the OCT results of four fingernails after exposure to these laser energies. Figure 1a–h represent the top view of the 3D OCT images and the representative cross-sectional images of four nails, respectively, which were obtained after laser exposures to energies of 50, 40, 30, and 20 mJ. Laser exposure induced MAZs as indicated by white arrows in Figure 1. Figure 1a–d demonstrate the increased size and penetration depth of MAZs corresponding to the increasing exposure energy. Based on the OCT results, the penetration depth and the diameter of the induced MAZ corresponding to exposure energy can be estimated. The average penetration depths of Figure 1a–d are 372, 321, 290, and 255 μm, respectively. Additionally, the average diameters of Figure 1a–d are 203, 183, 171, and 137 μm, respectively. The results show that an exposure energy of 50 mJ provides a deeper penetration depth while sparing the nail bed. Therefore, we chose 50 mJ as the optimal exposure energy to induce MAZ on the nails in the following experiments.

**Figure 2:**
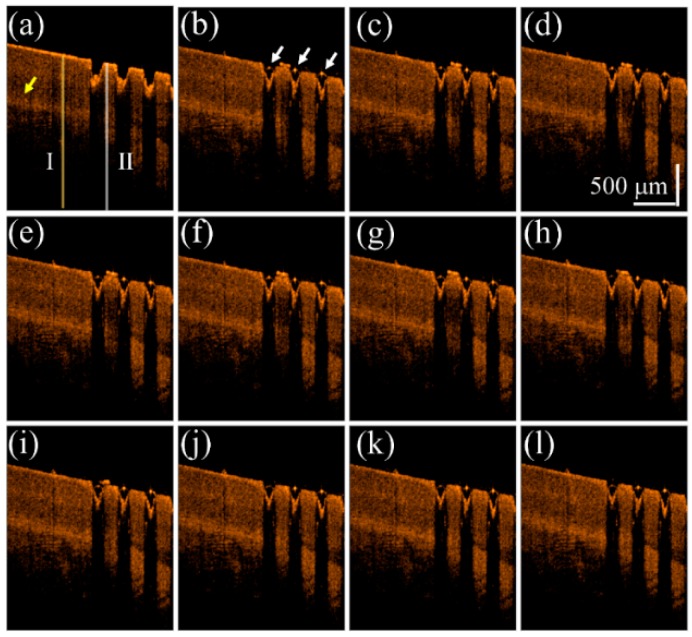
Time-series 2D OCT images obtained at the same location of the fingernail after 50 mJ fractional laser exposure. OCT images obtained (**a**) before the liquid drug application and at (**b**) 0 s; (**c**) 0.2 s; (**d**) 0.4 s; (**e**) 0.6 s; (**f**) 0.8 s; (**g**) 1.0 s; (**h**) 2.0 s; (**i**) 4.0 s; (**j**) 6.0 s; (**k**) 8.0 s; and (**l**) 10.0 s after the liquid drug application. The white arrows indicate that the stronger OCT backscattered signal resulted from the aggregation of drug particles. The yellow arrow indicates the nail bed. The yellow and white lines indicate the locations for estimation of the averaged A-scan profiles of the unexposed and treated regions.

To understand the influence on the OCT intensity of the unexposed and exposed nail regions after the drug application, a fingernail of one 22-year-old male volunteer was exposed to a fractional CO_2_ laser with an exposure energy of 50 mJ. In this case, only one-half of the fingernail was exposed, while the other half was spared. The finger was later fixed on the specially designed mount for motion reduction and scanned with OCT. We compare the difference of drug delivery between untreated nail and the laser-treated nail by treating both sides with liquid drug preparation and scanned with OCT. The scanning range covered both regions of the nail, and the changes before and after drug application were recorded. We analyze the intensity variation of OCT signal beneath the nail surface. A segmentation algorithm proposed in our previous study was used to explore the OCT signal beneath the nail surface [48,49]. Figure 2 shows the time-series 2D OCT images obtained at the same location of the fingernail. Figure 2a is the OCT image obtained before liquid drug application, where the left part is the untreated nail structure and the right part represents the laser-treated nail with MAZs. Figure 2b–l were obtained at various times after the liquid drug application. In Figure 2b, the strongly scattered spots, which are indicated by the white arrows, are a result of the aggregation of drug particles.

**Figure 3:**
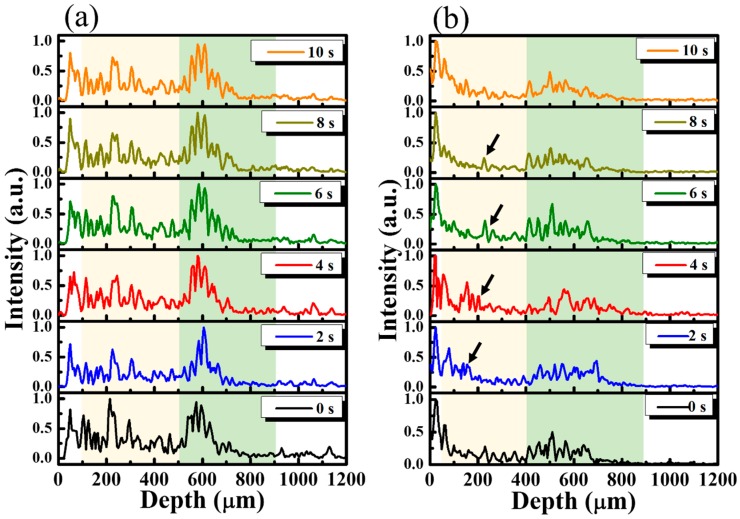
(**a**) Averaged A-scan profiles of the yellow line (the unexposed region) in Figure 2 and (**b**) the averaged A-scan profiles of the white line (the exposed region) in Figure 2 obtained at time points of 0, 2.0, 4.0, 6.0, 8.0, and 10.0 s after the drug application. The black arrows indicate the variation in OCT backscattered intensity after the drug application.

Figure 3 shows the averaged A-scan profiles of the unexposed and treated regions, as marked by the yellow and white lines in Figure 2a. Here, the A-scan represents a one-dimensional scan along the depth direction, representing the relationship between the backscattered intensity and the depth. For both lines, 11 adjacent A-scans, corresponding to a transverse range of 50 μm, were chosen for the acquisition of an averaged A-scan profile. Thus, Figure 3a represents the averaged A-scan profiles of the yellow line in Figure 2 obtained at 0, 2.0, 4.0, 6.0, 8.0, and 10.0 s after the drug application. In contrast, Figure 3b plots the averaged A-scan profiles of the white line in Figure 2 obtained at 0, 2.0, 4.0, 6.0, 8.0, and 10.0 s after the drug application. The yellowish region in Figure 3 represents the nail layer, and the greenish region indicates the tissue beneath the nail bed. In Figure 3a, the time-series of averaged A-scan profiles illustrate that there is no significant change in the backscattered intensity, especially in the yellowish region. In comparison to the results of Figure 3a, after the drug application, changes in the backscattered intensity of the yellowish region in Figure 3b was observed, marked by the black arrows. Our results show that the changes in OCT backscattered intensity can be used to identify the drug’s diffusion. However, because the vessels exist in the soft tissue of skin beneath the nail bed (the greenish region), which also result in OCT intensity variation, it is hard to tell whether these changes are due to the diffusion of drug particles or the motion of red blood cells in the soft tissue layer. Therefore, in this study, we focus on investigating the intensity variation of the nail plate.

According to the results in Figure 3, the diffusion of drug particles results in the variation of OCT backscattered intensity. Therefore, to quantitatively evaluate the intensity variation, the SV between the time-series OCT images was estimated. First, the OCT image obtained at the point of the drug application was used as a reference, and the OCT images obtained at various time points after the drug application were then individually compared with the reference image to acquire a corresponding SV image. Therefore, an SV image at time t after the drug application can be estimated as

(1) $$SV_{t_{n}}(x,z)=\frac{{\left\{I_{t_{0}}(x,z)-\frac{1}{2}[I_{t_{0}}(x,z)+I_{t_{n}}(x,z)]\right\}}^{2}+{\left\{I_{t_{n}}(x,z)-\frac{1}{2}[I_{t_{0}}(x,z)+I_{t_{n}}(x,z)]\right\}}^{2}}{2}$$

where x, z are the pixel locations in the transverse and longitudinal directions, respectively [43,44], and *t*_0_ and *t_n_* represent the start of the drug application and the nth time point after the drug application, respectively. In our previous study, although SV can be used to observe the diffusion of water through fingernails after fractional laser exposure, it was found to be difficult to further investigate the depth-resolved drug diffusion because of the shadowing effect resulting from particle diffusion [46]. Thus, to reduce the shadowing effect, Equation (1) can be revised as

(2) $$SVR_{t_{n}}(x,z)=SV_{t_{n}}(x,z)\times e^{\frac{1}{\gamma }\sum_{i=1}^{z}SV_{t_{n}}(x,i)}$$

where *γ* is an attenuation coefficient. To reject the contribution of speckle noise, we set the threshold SV value to 0.05, using the time-series 2D images to estimate the SV values before the drug application.

Subsequently, liquid and cream drugs were tested to study the feasibility of drug diffusion through MAZs. We repeat the same experiment protocol of Figure 2. First, the fingernails of one 24-year-old male volunteer were exposed to a fractional CO_2_ laser with an exposure energy of 50 mJ. During OCT scanning, the finger was fixed on the specially designed mount to reduce motion artifacts, and the same location of fingernail was continuously scanned by the OCT system to obtain a time series of 2D OCT images. The liquid drug preparation was then applied to the nail surface and the nail was continuously scanned for 60 s. To compare the intensity variance before and after the drug application, a 2D OCT image was obtained at the beginning of the drug application as the reference image, and time-series OCT images were recorded after the drug application to estimate the SV images. Finally, the OCT image and corresponding SV image at each time point were merged into an SV-OCT image.

**Figure 4:**
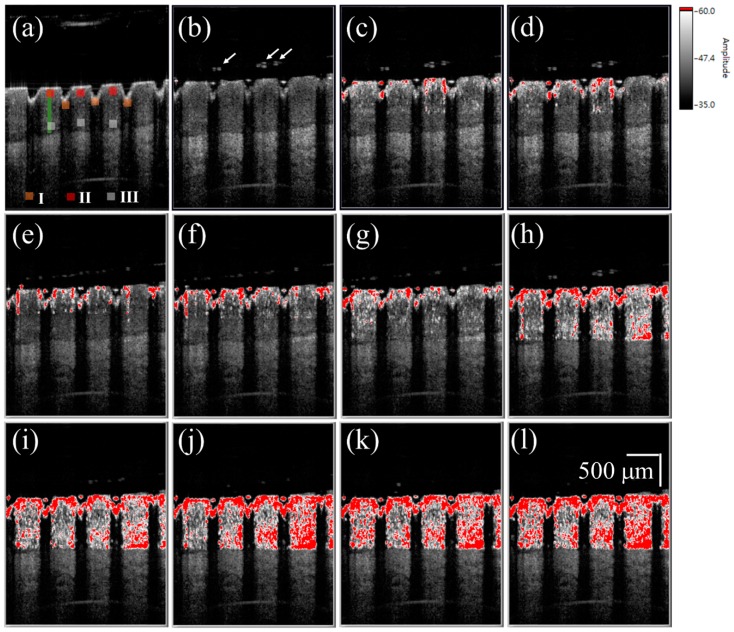
(**a**) 2D OCT image of the nail after 50 mJ laser exposure. Time-series SV-OCT images of the treated nail obtained after the liquid drug application at (**b**) 0 s; (**c**) 0.2 s; (**d**) 0.4 s; (**e**) 0.6 s; (**f**) 0.8 s; (**g**) 1.0 s; (**h**) 2.0 s; (**i**) 4.0 s; (**j**) 6.0 s; (**k**) 8.0 s; and (**l**) 10.0 s. The white arrows indicate the stronger backscattered signal, resulting from the drug particles. The scalar bar in (**l**) represents a length of 500 μm in length.

Figure 4a shows a 2D OCT image of the nail after fractional laser exposure with an exposure energy of 50 mJ, and Figure 4b–l represent time-series SV-OCT images obtained after the liquid drug application. To indicate the corresponding location of the SV signal in the nail, the OCT structural image and SV image were merged. The OCT structural intensity is shown in the gray scale, and the SV signal is shown in the red scale. Here, the occurrence of the SV signal indicates the location of intensity variance due to the moving particles, but the SV value is not proportional to the particle concentration. Strong backscattered spots, which are indicated by the white arrows in Figure 4b, moved with time, as shown in Figure 4b–l. These strong backscattered spots are a result of the aggregation of drug particles. The thickness of the liquid drug on the nail surface gives a redundant optical path difference, which will probably cause SV estimation errors. Therefore, a segmentation algorithm proposed in our previous study was performed before SV estimation [46]. Based on this segmentation algorithm, the nail surface can be detected, allowing the nail surfaces of the time-series OCT images to be realigned with the nail surface of the reference image. Since the blood flow in the soft tissue beneath the nail layer also causes time-dependent variations in OCT backscattered intensity, it is difficult to differentiate the SV contributions of the drug diffusion and the vessels in the soft tissue layer. Therefore, only SV signals in the nail structure are presented in this study; nevertheless, observations of the drug diffusion in the nail layer enable us to identify whether the drug particles have reached the nail bed. In Figure 4c, the SV signal began to occur around the boundaries of the induced MAZs, and the area of SV distribution then increased with time. After 10 s, the SV signal could be observed in the whole nail region.

**Figure 5:**
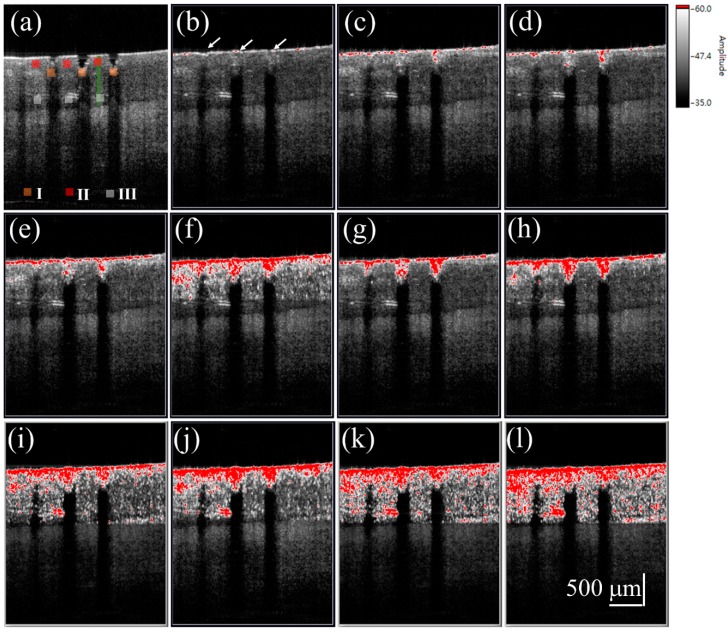
(**a**) 2D OCT image of the nail after fractional laser exposure with an exposure energy of 50 mJ. Time-series SV-OCT images of the nail obtained at (**b**) 0 s; (**c**) 0.2 s; (**d**) 0.4 s; (**e**) 0.6 s; (**f**) 0.8 s; (**g**) 1.0 s; (**h**) 2.0 s; (**i**) 4.0 s; (**j**) 6.0 s; (**k**) 8.0 s; and (**l**) 10.0 s after the cream drug application. The white arrows indicate that the MAZs were filled with the cream drug. The scalar bar in (**l**) represents a length of 500 μm in length.

To investigate the diffusion of the cream drug in the fingernail, the same finger in the experiment of Figure 4 was utilized again and the same experimental procedure was repeated on the next day of the liquid drug experiment. To avoid the accumulation of drug particles in the nail, the nails were immersed into the ultrasound cleaner to remove the unwanted depositions in the MAZs before each experiment. Additionally, in our method, we used the B-scan obtained in the beginning of the drug application as the reference image to estimate the SV. Therefore, the effect induced by the residual drug can be greatly reduced. The cream drug preparation was then rubbed onto the nail surface and simultaneously scanned by the OCT system for 60 s. Figure 5a shows a 2D OCT image of the treated nail obtained before the cream drug application, where the induced MAZ forms an inverted pyramid shape. Figure 5b–l are the time-series SV-OCT images obtained at various time points after the cream drug application. White color represents the tissue structure, and the red color indicates the existence of an SV signal. After the drug application, the cream drug preparation occupied the MAZs, causing a stronger backscattered intensity in the MAZ region. From Figure 5b–d, we can see that the SV signal only existed on the nail surface, and gradually occurred in the nail structure as time increased. After 10 s, SV was observed in the entire nail structure. This SV is a result of the time-dependent variation of OCT intensity due to the diffusion of drug particles. Again, only SV signals in the nail layer are presented. Additionally, a comparison of Figure 4 and Figure 5 suggests that the SV signals found in the MAZ of Figure 5 were absent in the MAZ of Figure 4. This is because the MAZs in Figure 5 were occupied by the cream drug particles. After applying the segmentation algorithm, an intact nail surface was found in Figure 5, and the MAZs in Figure 5 were included in the SV estimation. However, in the OCT images obtained from the experiment with the liquid drug after processing the segmentation algorithm, the MAZs were not included in the SV estimation.

**Figure 6:**
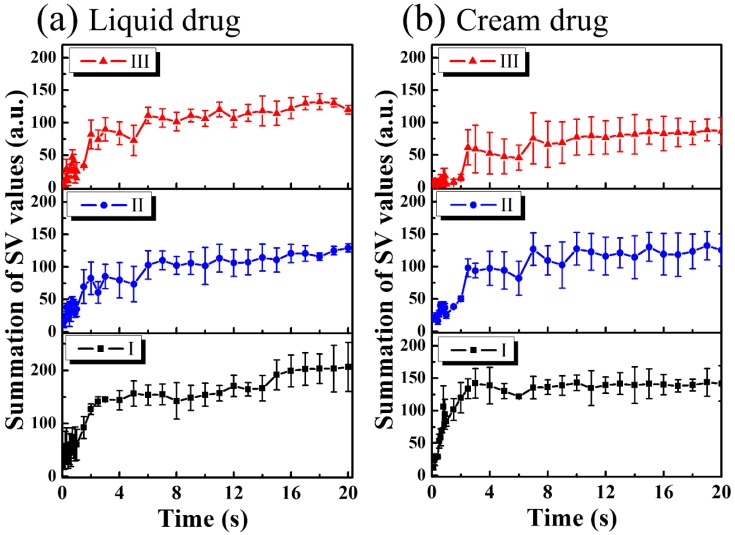
(**a**) Averaged summation of SV values of Regions I, II, and III indicated by the squares in Figure 4 as a function of time; (**b**) Averaged summation of SV values of Regions I, II, and III indicated by the squares in Figure 5 as a function of time.

For the study of drug particles diffusion behavior in nail layers, three regions (Regions I, II, and III in Figure 4 and Figure 5) were selected for analysis. Three orange squares located at the tip regions of the MAZs in Figure 4 and Figure 5 (Region I) were averaged, as were the three red squares located at the upper nail regions in Region II and the three white squares located in the middle of the two MAZs (Region III). For each region, the summation of the SV values of three colored square was averaged to acquire an averaged summation result at various time points. Figure 6a,b show the averaged summations of SV values of Regions I, II, and III in Figure 4 and Figure 5, respectively. From Figure 6a, we see that the averaged summation of the SV values in Region I increased after the drug application, reaching a saturation level after approximately 15 s. The results for Regions II and III in Figure 6a indicate that the averaged SV summation started to increase after 1 s. In comparison, Figure 6b shows the same trend for region I, but the summations only start to increase after 2 s in Regions II and III. Figure 6 show that MAZs effectively improve the drug diffusion through the nail layer. Three regions in each depth range (indicated by red, orange and white squares in Figure 4a and Figure 5a) are selected to estimate the average summation of SV values. The standard deviation of the three regions at the same depth range is shown in Figure 6.

## 4. Conclusions

In this study, we demonstrated that using a fractional ablative laser produces MAZ arrays on fingernails that facilitate drug delivery. However, the induced depth of photothermolysis is difficult to predict. Therefore, we used OCT for in vivo evaluation of photothermolysis on nail induced by the fractional CO_2_ laser. In addition, we propose a method here for in vivo observations of drug diffusion through the induced MAZs based on the evaluation of the time-dependent OCT intensity. In this study, the exposure energy for producing microthermal ablation zones in nails was set to be 50 mJ, which is the maximum output energy of the CO_2_ laser. From OCT scanning results, 50 mJ laser energy can induce an averaged penetration depth of more than 370 μm in nails, making drug particles easily penetrate the nail barrier and reach the skin tissue beneath the nail. These results suggest that OCT could serve as a potential tool for in vivo observations of drug diffusion.
